# Retrospective Observational Study of Atypical Winter Respiratory Illness Season Using Real-Time Syndromic Surveillance, England, 2014–15 

**DOI:** 10.3201/eid2311.161632

**Published:** 2017-11

**Authors:** Sue Smith, Roger Morbey, Richard G. Pebody, Thomas C. Hughes, Simon de Lusignan, F. Alex Yeates, Helen Thomas, Sarah J. O’Brien, Gillian E. Smith, Alex J. Elliot

**Affiliations:** Public Health England, Birmingham, UK (S. Smith, R. Morbey, G.E. Smith, A.J. Elliot);; Public Health England, London, UK (R.G. Pebody);; John Radcliffe Hospital, Oxford, UK (T.C. Hughes);; Royal College of Emergency Medicine, London (T.C. Hughes);; Royal College of General Practitioners, London (S. de Lusignan);; University of Surrey, Guildford, UK (S. de Lusignan);; Advanced Health and Care, Ashford, UK (F.A. Yeates);; National Health Service England, Leeds, UK (H. Thomas);; University of Liverpool, Liverpool, UK (S.J. O’Brien)

**Keywords:** influenza, respiratory syncytial virus, RSV, syndromic surveillance, asthma, severity, difficulty breathing, pneumonia, England, United Kingdom, respiratory infections, seasonal

## Abstract

During winter 2014–15, England experienced severe strains on acute health services. We investigated whether syndromic surveillance could contribute to understanding of the unusually high level of healthcare needs. We compared trends for several respiratory syndromic indicators from that winter to historical baselines. Cumulative and mean incidence rates were compared by winter and age group. All-age influenza-like illness was at expected levels; however, severe asthma and pneumonia levels were above those expected. Across several respiratory indicators, cumulative incidence rates during 2014–15 were similar to those of previous years, but higher for older persons; we saw increased rates of acute respiratory disease, including influenza like illness, severe asthma, and pneumonia, in the 65–74- and >75-year age groups. Age group–specific statistical algorithms may provide insights into the burden on health services and improve early warning in future winters.

Winter respiratory pathogens account for a large burden of community respiratory illness each year (*1**–**3*). Increased illness can result in pressures on hospitals, which can lead to local incidents where health services cannot meet patient demand, often leading to the closure of facilities (*4*). The elderly have been particularly implicated in driving hospital pressures due to increased admissions and increased lengths of stay (*5*). Although a wide range of viral and bacterial pathogens circulate each winter in the Northern Hemisphere, influenza viruses and respiratory syncytial virus (RSV) cause the greatest number of illnesses, especially among young children and the elderly. General practitioner (GP) consultations, emergency department (ED) visits, hospital admissions, and deaths all increase during periods of influenza and RSV activity: annual winter pressures on healthcare systems have been attributed in part to these pathogens (*6**–**10*). In the United Kingdom, seasonal influenza activity generally occurs during weeks 40–20 (October–May); the peak of activity is commonly December 25–January 1 or early January (*11*). RSV activity is typically more consistent, peaking during weeks 48–52 of each year, with evidence of a lag between the peaks among young children (week 48) and the elderly (week 52) (*12**,**13*). In the past, sentinel GP consultations for influenza-like illness (ILI) have been used as the standard for monitoring community-based influenza activity (*14**–**17*). These surveillance systems are now commonly interpreted as part of a suite of influenza-related monitoring systems.

Temporal analysis of GP ILI consultations in the United Kingdom has revealed a decreasing trend over the past 30 years, with particularly low to moderate activity recorded since winter 1999–2000 (*14*). There were 2 notable exceptions: the 2009–10 global pandemic, which saw substantial out-of-season summer and autumn activity (*18*); and winter 2010–11, when influenza activity reached levels higher than in any other winter since the millennium winter of 1999–2000 (*12**,**19*). To take such long-term trends into account, the United Kingdom, in common with many other countries in Europe, has implemented a Moving Epidemic Method to calculate the preepidemic threshold each season, with an approach that uses historical data to calculate the threshold (*20*). In more recent seasons, a spectrum of influenza surveillance systems, including virologic indicators, is used to determine levels of influenza activity in the community and health effects on the population, such as hospital admissions and excess deaths confirmed to be influenza related.

Public Health England (PHE) coordinates a surveillance program for influenza and other respiratory viruses including epidemiologic, virologic, and syndromic surveillance systems (*21**,**22*). Syndromic surveillance is the near real-time collection, analysis, interpretation, and dissemination of health-related data to enable the early identification of the impact (or absence of impact) of potential human or veterinary public health threats that require effective public health action (*23*). In England, PHE coordinates a national syndromic surveillance service that delivers daily real-time syndromic intelligence from several health data sources that have been described previously (*21*). Anonymized health data, including a range of symptoms and syndromes, are collected daily from several healthcare service providers across England. Counts of symptoms and syndromes are aggregated into several syndromic indicators (e.g., ILI, cough, vomiting, rash).

Moderate levels of influenza activity were seen in the community in the United Kingdom during 2014–15. Influenza A(H3N2) was the predominant virus circulating for most of the season, with influenza B circulating later in the season (*22*). The impact of A(H3N2) was predominantly seen in the elderly, with numerous outbreaks in residential care homes. Levels of all-cause excess deaths were also statistically much higher, at 5.4% excess deaths above the upper threshold (16,415 [95% CI 15,588–17,241] more excess deaths) than the last notable significant A(H3N2) season of 2008–09, which had 3.2% excess deaths above the upper threshold (10,438 [95% CI 9,977–10,964] more excess deaths) (*22*). However, in England there were reports of severe pressures within the National Health Service (NHS), particularly in emergency medicine, with more ED visits, longer waiting times in EDs, and a shortage of acute-care hospital beds (*4*). The aim of this study was to determine, retrospectively, whether syndromic surveillance could contribute intelligence toward identifying the underlying causes of these issues. We used national data collected routinely from a range of healthcare sources.

## Methods

### Study Period

PHE conducts enhanced surveillance of influenza and other respiratory viruses in the United Kingdom each winter from October (week 40) to May (week 20) (*22*). For this study, we used daily syndromic surveillance data extracted September 29, 2014–May 17, 2015. We extracted data from corresponding periods in 2 previous winters (2012–13, 2013–14) for comparison. To define a more specific period of intense winter activity during 2014–15, we selected a period of 5 weeks encompassing peak activity (*24*), corresponding to week 51 of 2014 through week 3 of 2015 (December 15, 2014–January 18, 2015).

### Syndromic Surveillance Data

Syndromic surveillance data used in this study included general practitioner consultations “in hours,” or during practitioners’ regular office hours (GPIH), and “out of hours” (GPOOH) services; ED visits from a sentinel network that is part of England’s Emergency Department Syndromic Surveillance System (EDSSS); and calls to the national NHS 111 telehealth service (*21**,**25**,**26*). We selected respiratory indicators from each data source, including ILI, upper and lower respiratory tract infection, acute respiratory infection, and pneumonia (Table 1). We captured, analyzed, and interpreted data contemporaneously with the study period by epidemiologic, statistical, and risk assessment processes (*27**,**28*).

**Table 1:** Syndromic surveillance data source and respiratory indicators used in study of an atypical winter respiratory illness season, England, 2014–15

Source	Indicator
GPIH	Upper respiratory tract infection; lower respiratory tract infections; influenza-like illness; “severe asthma”; pneumonia
GPOOH	Acute respiratory infection; influenza-like illness; asthma/wheeze/difficulty breathing
EDSSS	Acute respiratory infection; influenza-like illness; bronchitis; asthma/wheeze/difficulty breathing; pneumonia
NHS 111	Cold/flu; cough; difficulty breathing;

*EDSSS, Emergency Department Syndromic Surveillance System; GP, general practitioner; GPIH, GP in hours; GPOOH, GP out of hours; NHS 111, National Health Service telehealth service.

### Retrospective Descriptive Epidemiologic Analysis

We examined daily plots of syndromic surveillance data to establish trends over the winter period. We analyzed data according to the individual data source: incidence rates per 100,000 registered patient population (GPIH) or syndromic counts as a percentage of the total number of counts, where population coverage figures were unavailable (GPOOH, EDSSS, NHS 111). We stratified data by standard age groups (<1, 1–4, 5–14, 15–44, 45–64, 65–74, >75 years).

To compare respiratory activity during 2014–15 with previous winter seasons, we calculated baselines for each data source and syndromic indicator on the basis of available historic data for the data source. We also calculated weekly cumulative incidence plots for selected indicators and age groups for week 40 of 2014 through week 20 of 2015 and compared them to those for previous years.

### Statistical Analysis

To establish whether there were any statistically significant differences in respiratory syndromic activity between 2014–15 and other years, we used a nonparametric Mann-Whitney test to compare mean incidence rates between winter 2014–15 and the combined winter periods 2012–13 and 2013–14. We calculated the mean daily incidence of each respiratory indicator by age group for the period weeks 51–3 for each data source: the rate for 2014–15 was compared with that for the same period during the 2 preceding winters. We performed all statistical analyses with Stata version 13.1 (*29*).

## Results

### Trends in Respiratory Indicators 2014–15

**Figure 1:**
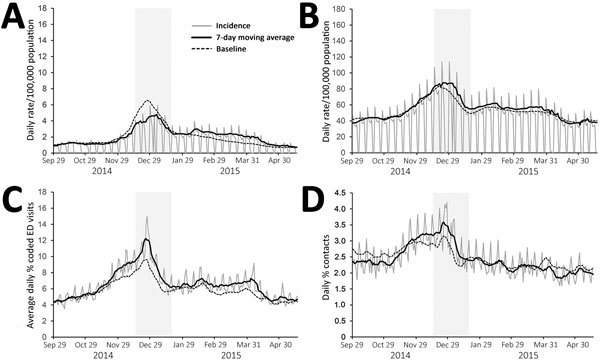
Daily incidence of acute respiratory indicators over winter 2014–15, England. A) General practitioner in hours (GPIH) influenza-like illness consultations; B) GPIH upper respiratory tract infection consultations; C) acute respiratory infection visits; D) general practitioner out of hours asthma/wheeze/difficulty breathing consultations. Vertical gray shaded area indicates period of peak winter activity (week 51 of 2014 through week 3 of 2015). ED, emergency department.

**Figure 2:**
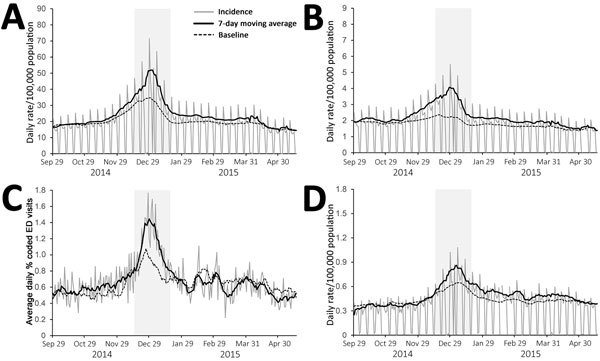
Daily incidence of severe respiratory indicators over winter 2014–15, England. A) General practitioner in hours (GPIH) lower respiratory tract infection consultations; B) GPIH severe asthma consultations; C) emergency department pneumonia visits; D) GPIH pneumonia consultations. Vertical gray shaded area indicates period of peak winter activity (week 51 of 2014 through week 3 of 2015). ED, emergency department.

The analysis of daily trends of syndromic indicators by all ages during the 2014–15 season revealed an increase in activity beginning in November 2014, with activity generally peaking between week 52 of 2014 and week 1 of 2015. There were, however, differences in the timing of peak activity: the earliest peak occurred in the GPIH, where the 7-day moving average of upper respiratory tract infection (URTI) consultations peaked on December 22, 2014 (Figure 1, panel B); the latest peak was also in the GPIH, where ILI and pneumonia consultations both peaked on January 5, 2015 (Figure 1, panel A; Figure 2, panel D).

Compared with historical baseline data, the activity of GPIH ILI (for all ages) was below seasonally expected levels (as demonstrated by historical baselines; Figure 1, panel A), whereas GPIH URTI, EDSSS acute respiratory illness, and GPOOH asthma/wheeze/difficulty breathing activity was just over baseline levels (Figure 1, panels B, C, and D). However, the levels of GPIH lower respiratory tract infection (LRTI) and severe asthma and of EDSSS pneumonia were considerably higher than expected during 2014–15 (Figure 2, panels A–C).

For all-age incidence, GPIH and EDSSS pneumonia, GPIH LRTI, EDSSS ILI, and GPIH severe asthma were significantly higher (p<0.05) during week 51 of 2014 through week 3 of 2015 than during the same period in the comparator seasons (Table 2; full results in Technical Appendix Table). Calls to the NHS 111 line for cold/ influenza were significantly higher (p = 0.009) in 2014–15, but due to the lack of historical data, we compared these data to those from 2013–14 only.

**Table 2:** Comparison of mean daily rate/percentage of selected respiratory indicators over weeks 51–3 between 2014–15 and previous 2 winters, England*

Syndromic surveillance			Age group, y							
Indicator	System	<1	1–4	5–14	15–44	45–64	65–74	>75	All ages
URTI	GPIH		0.178	0.270	0.540	0.086	0.066	**0.010**	**0.002**	0.066
ARI	GPOOH		0.807	0.713	0.903	0.713	0.391	0.111	0.050	0.540
	EDSSS		0.178	0.624	0.624	0.142	**0.037**	0.111	**0.005**	0.142
ILI	GPIH		0.391	0.221	0.462	0.178	0.142	**0.007**	**0.005**	0.142
	GPOOH		0.327	0.327	0.540	0.624	0.713	0.221	**0.037**	0.462
	EDSSS		0.480	0.312	0.061	**0.010**	0.178	**0.009**	**0.019**	**0.007**
Cold/influenza	NHS 111		0.410	**0.016**	**0.009**	**0.009**	**0.009**	**0.009**	**0.016**	**0.009**
Fever	NHS 111		0.602	0.251	**0.009**	0.076	0.602	0.175	0.602	0.175
LRTI	GPIH		0.462	0.270	0.327	0.111	0.086	**0.020**	**0.005**	**0.028**
Pneumonia	GPIH		**0.037**	0.178	0.462	**0.002**	**0.003**	**0.007**	**0.002**	**0.002**
	EDSSS		0.156	0.713	0.266	0.178	0.111	0.066	**0.020**	**0.028**
Cough	NHS 111		0.917	0.347	0.117	**0.047**	**0.028**	**0.028**	**0.047**	0.175
DB	NHS 111		0.347	0.465	0.465	0.175	0.117	0.076	**0.028**	0.175
A/W/DB	EDSSS		0.221	0.066	0.624	0.391	0.086	0.807	0.391	0.540
	GPOOH		0.624	1.000	0.462	0.111	0.111	**0.028**	**0.020**	0.178
Asthma	GPIH		0.167	0.903	0.178	**0.028**	**0.003**	**0.005**	**0.003**	**0.005**

*Activity during winter 2014–15 is compared to combined mean activity during previous 2 winters and results of Mann-Whitney test are presented as p values. Statistically significant (at 95% confidence level) results are in **bold**. ARI, acute respiratory tract infection; A/W/DB, asthma/wheeze/difficulty breathing; DB, difficulty breathing; EDSSS, emergency department syndromic surveillance system; GP, general practitioner; GPIH, GP in hours; GPOOH, GP out of hours; ILI, influenza-like illness; LRTI, lower respiratory tract infection; URTI, upper respiratory tract infection.

### Cumulative Incidence of Respiratory Indicators

**Figure 3:**
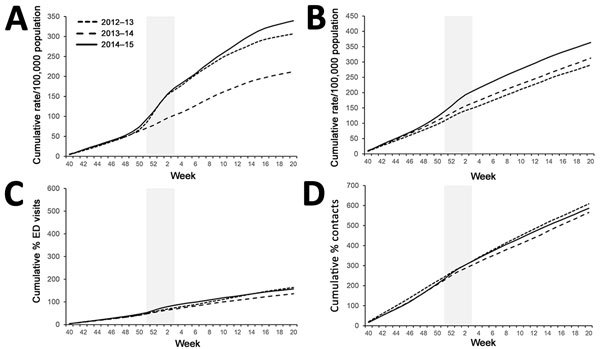
Weekly cumulative rates of selected respiratory indicators for all ages, England, winter 2014–15 compared with the previous 2 winters. A) General practitioner in hours (GPIH) influenza-like illness consultations; B) GPIH severe asthma consultations; C) ED pneumonia visits; D) general practitioner out of hours asthma/wheeze/difficulty breathing consultations. Vertical gray shaded area indicates period of peak winter activity (week 51 of 2014 through week 3 of 2015). ED, emergency department.

**Figure 4:**
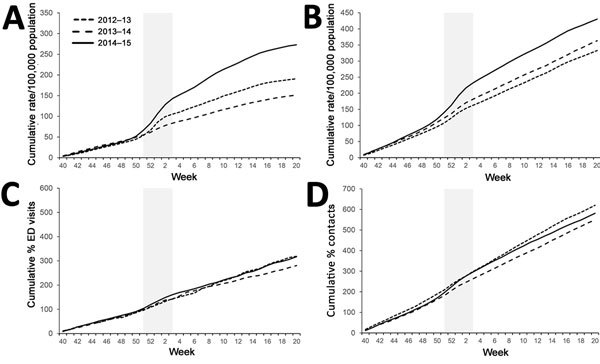
Weekly cumulative rates of selected respiratory indicators in the 65–74-year age group, England, winter 2014–15 compared with the previous 2 winters. A) General practitioner in hours (GPIH) influenza-like illness consultations; B) GPIH severe asthma consultations; C) ED pneumonia visits; D) general practitioner out of hours asthma/wheeze/difficulty breathing consultations. Vertical gray shaded area indicates period of peak winter activity (week 51 of 2014 through week 3 of 2015). ED, emergency department.

**Figure 5:**
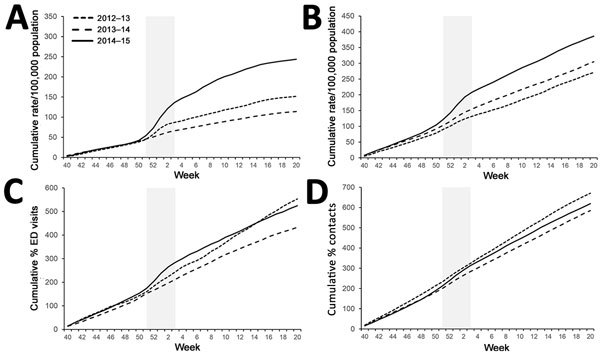
Weekly cumulative rates of selected respiratory indicators in the >75-year age group, England, winter 2014–15 compared with the previous 2 winters. A) General practitioner in hours (GPIH) influenza-like illness consultations; B) GPIH severe asthma consultations; C) ED pneumonia visits; D) general practitioner out of hours asthma/wheeze/difficulty breathing consultations. Vertical gray shaded area indicates period of peak winter activity (week 51 of 2014 through week 3 of 2015). ED, emergency department.

GPIH ILI all-age rates (Figure 3, panel A) during winter of 2012–13 and 2014–15 were similar with respect to the cumulative incidence slope, although the total cumulative incidence was greater for 2014–15 (307/100,000 population for 2012–13 and 339/100,000 population for 2014–15). Winter 2013–14 was demonstrably lower both in the slope of incidence and the final total cumulative incidence. GP ILI rates were noticeably higher in incidence for the 65–74- and >75-year age groups during 2014–15 (Figure 4, panel A; Figure 5, panel A); the cumulative rates for these age groups during 2014–15 started to diverge from the other winters during week 50 of 2014. Similarly, cumulative rates for GPIH severe asthma were comparable for all ages between 2014–15 and 2012–13 but were considerably higher for the 65–74- and >75-year age groups during 2014–15 (Figure 3, panel B; Figure 4, panel B; Figure 5, panel B); these rates were also higher for the 15–44- and 45–64-year age groups (data not shown).

The number of ED visits for pneumonia was similar between 2013–14 and 2014–15 across all ages and for the 65–74- and >75-year age groups, with activity levels higher than observed in 2012–13 (Figure 3, panel C; Figure 4, panel C; Figure 5, panel C). GPOOH consultations for asthma/wheeze/difficulty breathing were similar across all age groups and years (Figure 3, panel D; Figure 4, panel D; Figure 5, panel D).

For several key indicators, the statistical analysis confirmed the significant increases in older age groups during 2014–15. Of those indicators without evidence for statistically significant differences in the all-age incidence over weeks 51–3 between 2014–15 and other years, GPIH URTI was significantly higher in the 65–74- (p = 0.010) and >75-year (p = 0.002) age groups. Similarly, both GPIH ILI (65–74 years, p = 0.007; >75-year, p = 0.005) and GPOOH asthma/wheeze/difficulty breathing (65–74 years, p = 0.028; >75-year, p = 0.020) were significantly higher for older age groups (Table 2; Technical Appendix Table). 

## Discussion

We have undertaken a retrospective observational analysis of syndromic healthcare data that were monitored prospectively, on a daily basis, during winter 2014–15 as part of a national real-time syndromic surveillance service. Despite other indicators of influenza activity demonstrating moderate activity, although more severe in some instances (in particular for excess all-cause deaths and respiratory outbreaks in residential care home settings) (*22*), the national incidence of syndromic GP ILI for all ages and other indicators of community-based respiratory activity (e.g., URTI and ARI) were within seasonally expected levels. However, unusual activity was seen for certain syndromic conditions, including GPIH severe asthma and LRTI, as reported contemporaneously during the winter (*30*). At the time, it was unclear what was driving these observations; however, when these syndromic surveillance data were further stratified by age group and compared between years, it was apparent that increases in respiratory activity in the 65–74- and >75-year age groups were significantly greater than those seen during the previous 2 years. Our results support the hypothesis that a particular feature of this winter was the effect of respiratory infections in the elderly. The elderly have a higher risk for severe respiratory disease and secondary complications, resulting in excess hospital admissions (*5**,**8**,**9*).

Another important factor to take into account is that the dominant circulating strain in 2014–15 was influenza A(H3N2), a strain known to cause more severe disease in the elderly compared with other influenza subtypes, resulting in higher rates of disease and more severe outcomes in the elderly during seasons of predominant A(H3N2) circulation (*10**,**31**,**32*). Although 2014–15 was characterized by circulation of antigenically and genetically drifted influenza A(H3N2) and B viruses, end-of-season vaccine effectiveness estimates ultimately demonstrated an overall effectiveness of 34%, highlighting that, although effectiveness was suboptimal, the vaccine still provided important levels of protection to vulnerable populations (*33*). This finding was in line with recently published vaccine effectiveness estimates for this generation of inactivated vaccines (*34*).

The timing of influenza activity can be a contributing factor to pressures on health services. In the United Kingdom, peak respiratory admissions for bronchitis usually occur during week 52 of 1 year through week 2 of the next, coinciding with circulation of RSV (*12**,**13*). During winters in which both influenza and RSV, and therefore ILI and bronchitis, circulate concurrently, a higher-than-expected number of hospital admissions occur as the number of patients with respiratory illness is condensed into a shorter, more intense period (*35*). During winter 2014–15, influenza activity breached threshold levels during week 50 of 2014 and peaked in weeks 1 and 2 of 2015, overlapping with the peak of RSV activity. Although RSV is mainly known as a cause of bronchiolitis in children, it can also be an important cause of illness the elderly, in whom it can cause pneumonia and other LRTI (*3**,**36**,**37*).

The peak in GP consultations for severe asthma recorded during the winter of 2014–15 was nearly twice the baseline level. The increase preceded the increase in GP consultations for influenza and affected persons >15 years of age. The association between influenza infections and exacerbations of asthma has been documented through increased asthma hospital admissions in the elderly (*38*). However, the high number of GP consultations for severe asthma compared with other respiratory indicators during an influenza epidemic is unusual, and further work on the underlying causes of these excess consultations may be warranted.

Our study has several limitations. Syndromic surveillance is limited to monitoring and reporting on initial symptoms and provisional diagnoses; therefore, the results of this work cannot be directly linked or attributed to individual respiratory pathogens. However, previous comparisons of syndromic surveillance data with laboratory reports has shown that respiratory syndromes are sensitive to individual pathogens and can provide early warning of seasonal respiratory activity such as influenza and RSV (*39**–**43*).

In addition, several of the national syndromic surveillance data sources have been established since 2012 and therefore had limited historical data available for use in this study, which limits the comparison with a larger number of winters (*21**,**26*). Comparable GP surveillance data with extensive historical data confirm that influenza activity over the last decade has been relatively low, and therefore the comparison years were not atypical (*14*).

The number of winters included in this analysis was limited by the availability of surveillance data. Interseasonal differences in influenza vaccine effectiveness may also be a limiting factor when making comparisons; we therefore recommend future research to update our findings, including more winters and possibly stratifying by vaccine effectiveness.

We undertook this retrospective analysis to determine the relative burden of respiratory illness during the winter. However, to translate this research into the active public health service, it is important to understand how to apply these results to prospective data analysis. A common limitation of prospective analysis is delayed reporting, which can limit the usefulness of these data. However, the PHE syndromic surveillance data sources are all obtained in near real-time (daily), and the data are recorded consistently and completed at the time of the patient event.

Our findings support clinicians, health service managers, and public health bodies not relying on a single indicator of influenza activity to anticipate winter pressures on healthcare systems. GP consultations for ILI have historically been used as a key indicator of influenza activity in the community. However, persons with influenza can experience a wide range of clinical signs and symptoms (*6*). Our work, and that of others, continues to show that several clinical indicators, including ILI, LRTI, and severe asthma stratified by age, should be monitored routinely to identify expected sources of pressure.

Our work also highlights the importance of ensuring high levels of vaccine uptake in groups at higher risk for severe disease after influenza infection and also for children. The pediatric influenza vaccination program in England aims to protect the vaccinated children themselves and also, by reducing their rates of infection, reduce transmission in the population and thus indirectly protect those at higher risk for severe disease, such as the elderly and those with underlying clinical disease.

In the elderly, influenza accounts for more hospital admissions than RSV (*10**,**36**,**44*), but for those admitted to hospital with RSV, length of stay, rates of use of intensive care, and mortality rates can be similar to those for influenza patients (*36*). A growing body of evidence indicates that the effect of RSV in older patients is underestimated because they are not representatively sampled in the community. Whereas the contribution of RSV to increased hospital admissions in the winter of 2014–15 is uncertain, it may have contributed to the total burden placed on hospitals over that period. Improved testing and recognition of the effects of RSV by clinicians, particularly those working in geriatric medicine, might improve further understanding of this burden.

Underpinning each of the PHE national syndromic surveillance data sources are statistical algorithms that contemporaneously compare current data to historical data to determine whether activity is statistically higher and therefore requires public health action (*27*). The results of this study suggest that further development of these algorithms is needed to include age-specific statistics, that is, to monitor statistically significant activity in individual age groups rather than in all ages. This extended monitoring would offer a better means of providing early warning of increasing seasonal influenza severity and enable timelier public health action and interventions during future winters similar to 2014–15 (*45*). However, any increase in the daily workload required to monitor a large number of additional age-specific signals must be carefully considered. Using automated statistical algorithms to identify aberrations that require further epidemiologic interrogation can help minimize the impact on health systems while maintaining this extended monitoring (*27*).

The additional strain on the healthcare system during 2014–15 was experienced particularly in emergency medicine (*4*); the EDSSS was able to monitor trends at the national level, but the sentinel nature of this surveillance limited its usefulness for identifying and supporting local services. Future recruitment of additional EDs to the EDSSS across England, enabling expansion of this data source, would further facilitate use of local ED surveillance data.

Another area of potential future development is developing predictions and forecasting models to predict unusual activity in the elderly. Certain respiratory diagnoses, particularly in primary care and EDs, often peak in children with a lag before peaking in the elderly (*12**,**43*). It has also been shown that within primary care, elderly patients seek treatment later in the disease episode when symptoms have deteriorated (*46*). Using these observations, public health epidemiologists could monitor early incidence in children to model and predict unusual activity in the elderly.

Technical Appendix Additional information about respiratory illnesses in England during winter 2014–15.
