# Supplementary material for: Retrospective Observational Study of Atypical Winter Respiratory Illness Season Using Real-Time Syndromic Surveillance, England, 2014–15
Source: Emerg Infect Dis. 2017 Nov;23(11):1834–42. doi: 10.3201/eid2311.161632 (PMC5652417; doi:10.3201/eid2311.161632)
Supplement: Technical Appendix — Additional information about respiratory illnesses in England during winter 2014–15. [file 16-1632-Techapp-s1.pdf]

Article DOI: https://doi.org/10.3201/eid2311.161632

Retrospective Observational Study of Atypical Winter Respiratory Illness Season Using Real-Time Syndromic Surveillance, England, 2014–15

Technical Appendix

Technical Appendix Table. Mean daily rate/percentage of selected respiratory indicators (over weeks 51–03) for individual winters by age group. Activity during winter 2014–15 is compared to combined mean activity during previous 2 winters and results of Mann Whitney test are presented.*

			Age group (y)							
Indicator†	System‡	Winter	<1	1–4	5–14	15–44	45–64	65–74	75+	All ages
URTI	GPIH	2012–13	318.02	208.54	101.24	54.49	51.49	40.66	36.16	66.70
		2013–14	393.08	210.56	58.37	45.64	44.11	43.84	41.90	57.97
		2014–15	429.38	253.74	90.31	59.31	59.72	60.54	68.39	77.00
		p§	0.178	0.270	0.540	0.086	0.066	**0.010**	**0.002**	0.066
ARI	GPOOH	2012–13	32.93	41.61	39.18	22.21	19.63	14.16	14.36	24.56
		2013–14	34.91	40.12	30.48	17.18	13.87	11.47	12.05	19.93
		2014–15	34.33	40.36	33.93	20.55	18.68	16.65	17.79	23.36
		p§	0.807	0.713	0.903	0.713	0.391	0.111	0.050	0.540
ARI	EDSSS	2012–13	30.51	25.99	12.53	4.82	4.56	5.34	7.95	8.20
		2013–14	36.10	24.24	7.27	3.98	3.72	4.92	6.54	6.83
		2014–15	37.96	26.64	11.63	5.43	5.30	6.86	10.63	9.15
		p§	0.178	0.624	0.624	0.142	**0.037**	0.111	**0.005**	0.142
ILI	GPIH	2012–13	1.12	2.32	3.23	4.33	5.27	2.42	1.95	3.96
		2013–14	1.63	1.32	0.64	1.79	1.94	1.35	1.03	1.57
		2014–15	1.64	2.22	2.17	4.10	4.85	3.64	3.77	3.89
		p§	0.391	0.221	0.462	0.178	0.142	**0.007**	**0.005**	0.142
ILI	GPOOH	2012–13	0.34	0.41	0.86	1.21	1.14	0.47	0.22	0.82
		2013–14	0.21	0.22	0.24	0.43	0.34	0.18	0.09	0.29
		2014–15	0.33	0.34	0.52	0.85	0.71	0.47	0.27	0.59
		p§	0.327	0.327	0.540	0.624	0.713	0.221	**0.037**	0.462
ILI	EDSSS	2012–13	0.00	0.07	0.10	0.24	0.07	0.00	0.02	0.12
		2013–14	0.04	0.10	0.05	0.14	0.08	0.04	0.00	0.09
		2014–15	0.00	0.04	0.20	0.34	0.15	0.14	0.08	0.21
		p§	0.480	0.312	0.061	**0.010**	0.178	**0.009**	**0.019**	**0.007**
Cold/influenza	NHS 111	2012–13¶	–	–	–	–	–	–	–	–
		2013–14	0.01	1.68	0.48	0.70	0.72	0.57	0.34	0.69
		2014–15	0.00	2.60	1.25	1.46	1.52	1.24	0.73	1.35
		p	0.410	**0.016**	**0.009**	**0.009**	**0.009**	**0.009**	**0.016**	**0.009**
Fever	NHS 111	2012–13¶	–	–	–	–	–	–	–	–
		2013–14	4.81	6.20	1.38	0.24	0.29	0.43	0.36	1.31
		2014–15	4.81	6.92	2.17	0.30	0.27	0.36	0.33	1.44
		p	0.602	0.251	**0.009**	0.076	0.602	0.175	0.602	0.175
LRTI	GPIH	2012–13	100.24	43.20	15.02	16.54	38.31	53.64	81.87	32.39
		2013–14	132.86	46.49	8.12	12.54	30.52	49.95	80.84	28.04
		2014–15	133.56	50.26	13.65	17.64	42.96	73.19	135.58	40.37
		p§	0.462	0.270	0.327	0.111	0.086	**0.020**	**0.005**	**0.028**
Pneumonia	GPIH	2012–13	0.17	0.23	0.15	0.18	0.40	0.84	1.61	0.40
		2013–14	0.21	0.44	0.07	0.19	0.45	0.83	2.19	0.47
		2014–15	0.42	0.43	0.13	0.27	0.64	1.26	3.60	0.70
		p§	**0.037**	0.178	0.462	**0.002**	**0.003**	**0.007**	**0.002**	**0.002**
Pneumonia	EDSSS	2012–13	0.15	0.76	0.29	0.31	0.70	1.59	2.88	0.89
		2013–14	0.25	0.40	0.12	0.30	0.86	1.60	2.23	0.77
		2014–15	0.06	0.44	0.13	0.42	0.93	1.99	3.73	1.12
		p§	0.156	0.713	0.266	0.178	0.111	0.066	**0.020**	**0.028**
Cough	NHS 111	12–13¶	–	–	–	–	–	–	–	–

Indicator†	System‡	Winter	Age group (y)							
			<1	1–4	5–14	15–44	45–64	65–74	75+	All ages
		13–14	15.04	12.62	5.15	2.16	3.15	3.43	3.20	4.86
		14–15	15.42	14.06	8.38	3.71	5.58	6.23	5.58	6.79
		p	0.917	0.347	0.117	**0.047**	**0.028**	**0.028**	**0.047**	0.175
DB	NHS 111	12–13¶	–	–	–	–	–	–	–	–
		13–14	9.94	6.31	2.59	1.88	3.07	4.34	4.68	3.80
		14–15	8.98	5.77	2.86	2.38	3.78	5.30	5.90	4.27
		p	0.347	0.465	0.465	0.175	0.117	0.076	**0.028**	0.175
A/W/DB	EDSSS	2012–13	2.77	3.97	2.07	1.29	1.63	1.91	2.05	1.80
		2013–14	2.02	3.03	2.35	1.18	1.36	1.29	1.28	1.46
		2014–15	1.84	2.43	2.38	1.51	1.85	1.70	1.70	1.75
		p§	0.221	0.066	0.624	0.391	0.086	0.807	0.391	0.540
A/W/DB	GPOOH	2012–13	4.05	3.40	2.63	2.11	3.00	3.13	3.19	2.80
		2013–14	4.27	3.82	3.06	1.93	2.58	2.86	2.95	2.72
		2014–15	3.98	3.55	3.12	2.51	3.30	3.61	3.58	3.16
		p§	0.624	1.000	0.462	0.111	0.111	**0.028**	**0.020**	0.178
Asthma	GPIH	2012–13	0.10	1.29	1.68	1.77	2.76	2.70	2.13	2.08
		2013–14	0.03	1.31	1.80	1.87	3.06	2.91	2.52	2.26
		2014–15	0.01	1.30	2.33	2.67	4.61	4.54	4.17	3.31
		p§	0.167	0.903	0.178	**0.028**	**0.003**	**0.005**	**0.003**	**0.005**

*Statistically significant (at 95% confidence level) results are presented in **bold**.
†URTI – upper respiratory tract infection; ARI – acute respiratory tract infection; ILI – influenza-like illness; LRTI – lower respiratory tract infection; DB – difficulty breathing; A/W/DB- asthma/wheeze/difficulty breathing.
‡GPIH, general practice in hours; GPOOH, general practice out of hours; EDSSS, Emergency Department Syndromic Surveillance System.
§p value for difference between mean daily rate for 2014–15 over weeks 51–03 compared to the same weeks for 2012–13 and 2013–14 combined.
¶Data not available for 2012–13 for the NHS 111 system so can only compare 2014–15 with 2013–14.
